# QbD Based Media Development for the Production of Fab Fragments in *E. coli*

**DOI:** 10.3390/bioengineering6020029

**Published:** 2019-03-28

**Authors:** Deepak Kumar, Jyoti Batra, Claire Komives, Anurag S. Rathore

**Affiliations:** 1 Department of Chemical Engineering, Indian Institute of Technology, Hauz Khas 110016, India; dpakk10@gmail.com (D.K.); batrajyoti123@gmail.com (J.B.); 2 Department of Biomedical, Chemical and Materials Engineering, San Jose State University, San Jose, CA 95192, USA; claire.komives@sjsu.edu

**Keywords:** quality by design, Ranibizumab, design of experiments, media development

## Abstract

Ranibizumab is a biotherapeutic Fab fragment used for the treatment of age-related macular degeneration and macular oedema. It is currently expressed in the gram-negative bacterium, *Escherichia coli*. However, low expression levels result in a high manufacturing cost. The protein expression can be increased by manipulating nutritional requirements (carbon source, nitrogen source, buffering agent), process parameters (pH, inducer concentration, agitation, temperature), and the genetic make-up of the producing strain. Further, understanding the impact of these factors on product quality is a requirement as per the principles of Quality by Design (QbD). In this paper, we examine the effect of various media components and process parameters on the expression level and quality of the biotherapeutic. First, risk analysis was performed to shortlist different media components based on the literature. Next, experiments were performed to screen these components. Eight components were identified for further investigation and were examined for their effect and interactions using a Fractional Factorial experimental design. Sucrose, biotin, and pantothenate were found to have the maximum effect during Fab production. Furthermore, cyanocobalamin glutathione and biotin-glutathione were the most significant interactions observed. Product identification was performed with Liquid Chromatography–Mass Spectrometry (LC-MS), the expression level was quantified using Bio-layer Interferometry, Reverse Phase-HPLC, and SDS-PAGE, and product quality were measured by RP-HPLC. Overall, a five-fold enhancement of the target protein titer was obtained (from 5 mg/L to 25 mg/L) using the screened medium components vis-a-vis the basal medium, thereby demonstrating the efficacy of the systematic approach purported by QbD.

## 1. Introduction

Monoclonal antibodies (mAb) and their derivatives such as antibody fragments (Fab) and single chain fragments (scFv) dominate the biopharmaceutical industry with their demonstrated effectiveness for the treatment of various life-threatening diseases [1]. Recombinant antibody fragments have considerable commercial potential as these molecules possess a similar antigen binding affinity as that of the parent molecule with the added advantage of simplified bioprocessing and lower cost because of production in microbial hosts [2]. Ranibizumab is a recombinant humanized IgG1 kappa isotype monoclonal antibody fragment designed for intraocular use [3]. It has been approved to treat the “wet” type of age-related macular degeneration (AMD), a common form of age-related vision loss [4]. However, lower expression levels render the manufacturing uneconomical [4,5].

Biopharmaceutical process development usually faces a plethora of challenges due to the complex and heterogeneous nature of biotherapeutic products because of which their complete analytical characterization is not possible [6]. Media development in upstream processing (USP) is one of the major challenges due to its significant impact on process productivity, cell growth, and product quality. Optimization of media, however, remains a non-trivial task due to the significant diversity of media components and interactions between components and metabolic pathways.

Traditionally, complex media has been popular due to favourable costing and ease of preparation. However, lack of knowledge of its composition results in batch to batch variability affecting process performance as well as product quality [7]. As a result, the use of defined media is steadily increasing as all the components are well characterized and quantified and, as a result, its use allows for a simpler purification process with improved reproducibility. In particular, this offers a significant advantage when process control is to be achieved by means of controlling the growth rate [8]. A variant of a defined medium is a minimal medium that contains components that are specifically required for growth including carbon sources (e.g., glucose), inorganic salts, trace elements, vitamins, and amino acids [8].

Media components such as amino acids and sugars act as the building blocks of proteins and nucleotide precursors, respectively. Supplements such as micronutrients and enzyme co-factors further accelerate cell growth. Excess medium nutrients may lead to the generation of unwanted metabolic by-products, resulting in reduced cell viability and product yield [9]. High concentration of carbon source has been shown to result in the diversion of the fermentative pathway to formation of acetic acid whereas lower than optimal concentrations can result in cell starvation, resulting in cell stress and favouring the propagation of the plasmid-free population and lower protein production [10]. Consequently, a careful balance is required with respect to concentrations of the various media components and their interactions with process parameters such as pH, temperature, and agitation for achieving optimal protein production [11]. 

The one factor at a time (OFAT) approach serves as the conventional strategy for design, selection, and optimization of fermentation media. However, the biopharmaceutical research community has reached a consensus that for most applications, OFAT is a laborious and time-consuming exercise and, most importantly fails, to unravel the synergistic interactions of components. Nonetheless, if a large number of factors are to be examined, OFAT can be useful for screening them prior to performing design of experiments (DOE) studies. All these efforts are aimed at gaining a deeper understanding of the process and its effects on the product, in line with principles of Quality by Design (QbD) [12,13,14]. According to the ICH Q8 guidelines, QbD is a systematic approach which emphasises the building product quality by better understanding the process itself [15]. Increased process knowledge can enhance process development by delivering an optimized and robust process [16]. 

Optimization of fermentation media is influenced by the nature of the cell line as well as the therapeutic protein under consideration. Careful optimization of the medium components and process parameters can minimize the level of process-related impurities in the fermentation broth and, thereby, reduce the burden of downstream processing, significantly altering the overall process economics. The rationale behind media optimization is enhancing the final yield of the target product by identifying the most significant nutrients and, thereafter, optimizing their concentration. In order to assess the genetic and metabolic burden of the plasmid in recombinant cells, various growth media need to be screened, finally narrowing it down to the optimal one [17]. Each nutrient and media component has a unique role to play depending on the cellular environment. Thus, examining these individual components, as well as their interactions, is of foremost importance. This helps in establishing a platform process in the early stages of drug discovery under the QbD paradigm, thereby expediting the overall bioprocess development.

The objective of this study was to examine the role of the various media components and thereby create an optimal medium based on QbD principles that would give the high cellular growth of *E. coli* together with the high productivity of the Fab product under consideration. First, different basal media (King’s medium [18], Cossins medium [19], Sletta medium [20], Zhang medium [21], Champion medium [22], and Riesenberg medium [23]) were shortlisted based on the literature review and screened for the application at hand. Next, experiments were performed to screen a variety of media additives and then, based on the results, eight components were identified for further investigation. Finally, eight components were shortlisted and further examined for their effect and for their interactions using a Fractional Factorial experimental design. To our knowledge, this is the first such report involving the optimization of the production media for the production of a Fab biotherapeutic.

## 2. Materials and Methods

### 2.1. Strain and Genetic Construct

The plasmid was purchased from DNA2.0 (currently ATUM, Newark, CA, USA). The protein sequence for the heavy and light chains of Ranibizumab was obtained from the Drug Bank (DB01270). The gene sequence was optimized for *E. coli*. The sequence was submitted to the NCBI GenBank and the accession number is MH507507. The plasmid chosen was pD881, a low copy number plasmid with the kanamycin resistance gene for selection. The rhamnose promoter (rhaBAD) was chosen for an inducible system. A strong ribosome binding sequence was chosen. Two different secretion sequences were selected for the heavy and light chain genes, which were the mal and pelB sequences, respectively. The heavy chain was placed upstream from the light chain and the cloning process omitted the methionine upstream of the heavy chain gene for fidelity to the original sequence. BL21 (DE3) *E. coli* cells were transformed with the plasmid for good expression levels and the deletion of proteases.

### 2.2. Chemicals and Media

All chemicals were of the analytical grade and obtained from Sigma Aldrich (St. Louis, MI, USA). Shake flask cultures were performed with Luria Bertani (LB) broth containing 30 µg/mL of Kanamycin. Frozen stocks of transformed *E. coli* were prepared from LB overnight cultures and maintained at −80 °C with 10% glycerol.

### 2.3. Screening of Growth Media

Initially, different growth media including King’s medium [18], Cossins medium [19], Sletta medium [20], Zhang medium [21], Champion medium [22], and Riesenberg medium [23] were screened for maximal cell concentration (Table 1). Baffled shake flasks with a capacity of 250 mL containing 50 mL of media were used for bacterial cell culture. Cells were cultivated at 30 °C and 200 rpm and the culture samples were taken at regular intervals of 1 h and the absorbance (OD) was measured at 600 nm. All experiments were performed in duplicate.

### 2.4. Purification of Fab Using Capto L Resin

To confirm the presence of the Fab protein, the concentrated sample was loaded onto Capto L column pre-equilibrated with equilibration buffer (20 mM Sodium Phosphate, 150 mM NaCl, pH 7.2) at a flow rate of 0.2 mL/min. The Fab fragment was eluted from the column via isocratic elution using a buffer (0.1 M Glycine, pH 3.0) at a flow rate of 0.5 mL/min with a dynamic binding capacity (DBC) of 18 mg/mL and a residence time of 4 min. The pH corresponding to the elution peak of the Fab was determined to be 3.25 based on a decreasing pH linear gradient elution from 6.0 to 2.5. The flow-through and elute fractions were stored at 4 °C for further analysis.

### 2.5. Liquid Chromatography-Mass Spectrometry (LC-MS) of Intact Fab

The LC-MS of the Fab was performed to determine its molecular mass. RP-HPLC was conducted using a Zodiacsil column (150 × 4.6 mm) and an Agilent 1260 Infinity Bio-inert Quaternary LC system coupled to an Agilent 6230 electrospray ionization-time of flight-mass spectrometer (ESI-TOF-MS) instrument. Gradient elution was carried out with 0.1% (*v*/*v*) TFA in MilliQ water (A) and 0.1% (*v*/*v*) TFA in acetonitrile (B) at 0.5 mL/min and 70 °C from 45% B in 5 min and followed by 45 to 100% in 25 min. MS was calibrated in the positive ion mode with MS spectra recorded for m/z 500–3200. The mass spectrum obtained was deconvoluted using the maximum entropy algorithm in the Agilent MassHunter Qualitative Analysis and BioConfirm Intact Protein workflow. MS source parameters included a gas temperature of 250 °C, a gas flow rate of 10 L/min, and a nebulizer pressure of 40 psig. Scan source parameters were as follows: Vcap: 4500, Fragmentor: 300, Skimmer: 65 and Octopole RFPeak of 750.

### 2.6. Screening of Process Parameters

Various physicochemical parameters including pH (6.0–8.0), temperature (20–37 °C), agitation (150–250 rpm), and inducer concentration (25–150 mM) were screened for their effect on Fab production. All experiments were performed in duplicate.

### 2.7. Screening of Various Medium Components Using OFAT Approach

Various media components including glycine (0.2%), ammonium sulphate (0.4%), di-sodium succinate (1%), L-proline (0.2 g/L), L-isoleucine (0.2 g/L), ferrous chloride (FeCl_2_) (2.7%), potassium sulphate (0.18%), aluminium sulphate (2 g/L), biotin (1 mg/L), di-potassium hydrogen phosphate (K_2_HPO_4_) (0.12%), calcium sulphate (0.1%), yeast extract (1%), sodium chloride (1%), ammonium chloride (0.2%), peptone (2%), pantothenate (1 mg/L), nickel sulphate (25 g/L), cobalt sulphate (0.75 g/L), cyanocobalamin (1.4 mg/L), sodium ethylenediaminetetraacetic acid (EDTA) (10.5 mg/L), sodium bicarbonate (1%), ferrous sulphate (FeSO_4_) (0.1%), glutathione (10 mM), sucrose (1%), and zinc chloride (0.2%) were screened for their effect on Fab production. The Riesenberg medium was used as the basal medium. Bacterial cells were cultivated at 30 °C and 200 rpm in baffled shake flasks with a capacity of 250 mL. All experiments were performed in duplicate. 

### 2.8. Fractional Factorial Design Based Two-Level Screening of Selected Medium Components

A fractional factorial resolution for a design of experiments (DOE) study was performed with 2-factor interactions [24]. Statistical modelling was performed and an empirical correlation was developed between the media constituents and product titre using the JMP software (SAS Institute, Cary, NC, USA). The media additives selected were Potassium hypophosphate (K_2_HPO_4_), biotin, glutathione, sucrose, potassium sulphate, calcium sulphate, pantothenate, and cyanocobalamin. Each additive was screened at two concentrations (high and low) (Table 2). All experiments were performed in duplicate. 

### 2.9. Periplasmic Protein Extraction

The periplasmic protein fraction of *E. coli* was extracted by cold osmotic shock [25]. Briefly, the harvested bacterial cells were gently suspended in Tris-EDTA-Sucrose (TES) buffer (50 mM Tris buffer pH 7.4, 1 mM EDTA and 20% sucrose *w*/*v*) and were incubated on ice for 30 min. The periplasmic fraction was separated by centrifuging the cells at 3250 g for 10 min. The supernatant containing periplasmic protein fraction was concentrated using centrifugal concentrators.

### 2.10. Fab Quantification by SDS PAGE 

The protein concentration was determined by the Bradford method using bovine serum albumin as the standard [26]. Absorbance was measured at 595 nm. Protein fractions were analyzed by SDS-PAGE using 12.5% acrylamide gel. Gels were rinsed three times with Milli Q water, stained with silver nitrate, and analyzed with the Image J Software.

### 2.11. Fab Quantification by Reversed-Phase High-Performance Liquid Chromatography (RP-HPLC)

Samples were analyzed by RP-HPLC on Ultimate 3000 (Dionex, Thermo Scientific) using the Zodiacsil column (150 × 4.6 mm) operated at 70 °C and with a flow rate of 0.5 mL/min. Mobile phase A consisted of 0.1% TFA in Milli Q water and mobile phase B consisted of 0.1% TFA in acetonitrile. Equilibration of the column was achieved using 45% B for 5 min. Elution was performed using a linear gradient of 45–100% B in 25 min. The column was regenerated using 45% B for 15 min. Protein detection was performed by UV absorption at 214 nm. Purity was determined based on the area of the peaks in the chromatogram. 

### 2.12. Fab Quantification by BLI

Protein L possesses a high affinity for the kappa light chain of antibodies and antibody fragments. The immunoglobulin binding of IgG to the Protein L does not interfere with its antigen-binding property [27]. This binding affinity on Protein L biosensors has been used to determine the antibody or antibody fragment moiety concentration as the interference pattern of light reflected from the biosensor surface changes upon binding, allowing the association and dissociation events to be monitored in real time [28]. Octet RED96 (Fortebio, Pall Life Sciences, CA, USA) was used for the quantification of antibody fragment using Protein L Biosensors (ForteBio part no, 18-5085, Pall Life Sciences, Port Washington, NY, USA). The standard curve was prepared based on the purified Fab molecule at concentrations of 5 µg/mL, 10 µg/mL, 15 µg/mL, and 25 µg/mL. To rule out any possibility of variability with wells, standard curves were repeated in different well locations in three independent experiments. All samples to be analyzed were diluted in the sample diluent. The hydration of the protein L sensor was performed in phosphate buffer saline (PBS) at pH 7.4. Regeneration was performed in 10 mM glycine (pH 1.5) and the neutralization buffer was kept identical to the hydration buffer. The control/samples (200 µL/well) and the hydration solution were placed in the wells corresponding to the position of the biosensor to be used in the analysis and were hydrated for 10 min prior to starting the experiment. Subsequently, samples were quantified at 130 g for 300 s [28]. All experiments were performed at 30 °C. The assay was performed in the basic quantitation with the regeneration assay format of the Octet data acquisition software. Data analysis was performed using the Octet Data Analysis Software version 7.1 from Pall Life Sciences. The acquired data was corrected by subtracting the blank matrix from the sample matrix which was analyzed using the initial slope binding rate equation.

## 3. Results

### 3.1. Screening of Basal Growth Media

**Figure 1:**
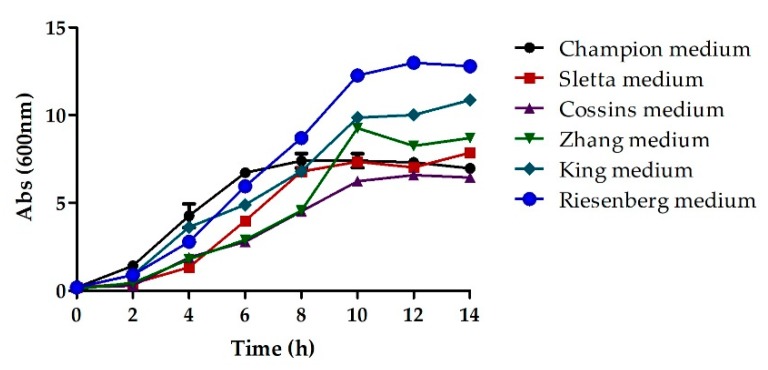
The cell density as a function of time plotted for different basal media.

The selection of growth media is crucial to attaining the desired productivity and quality [29,30]. Previous studies have shown that the usage of chemically defined media helps achieve better productivity and reproducibility in a cost-efficient manner [9,31,32]. This approach results in the maximal conversion of the substrates into the product with the simultaneous elimination of undesirable components that would otherwise interfere with purification. In order to optimize the media, the concentration and nature of the medium components were examined via shake flask experimentation and their impact on growth rate and cell density (Biomass) was examined by comparison of optical density. High turbidity was observed in the flask containing the Champion medium and an optical density (OD @600 nm) of 8 was observed after 10 h of cultures. The high turbidity could be attributed to the high concentration of metal ions present in this medium. The use of Sletta medium yielded a maximum OD of 7.34 after 10 h of cultures whereas that with Cossins medium was 6.23, implying low growth. A higher OD of 10 was observed with both Zhang and King medium after 10 h. The highest OD after 10 h of cultures was that of 12.26 with the Riesenberg medium (Figure 1). Thus, the Riesenberg medium was selected as the basal medium for all subsequent experiments.

### 3.2. Confirmation of Product Formation

**Figure 2:**
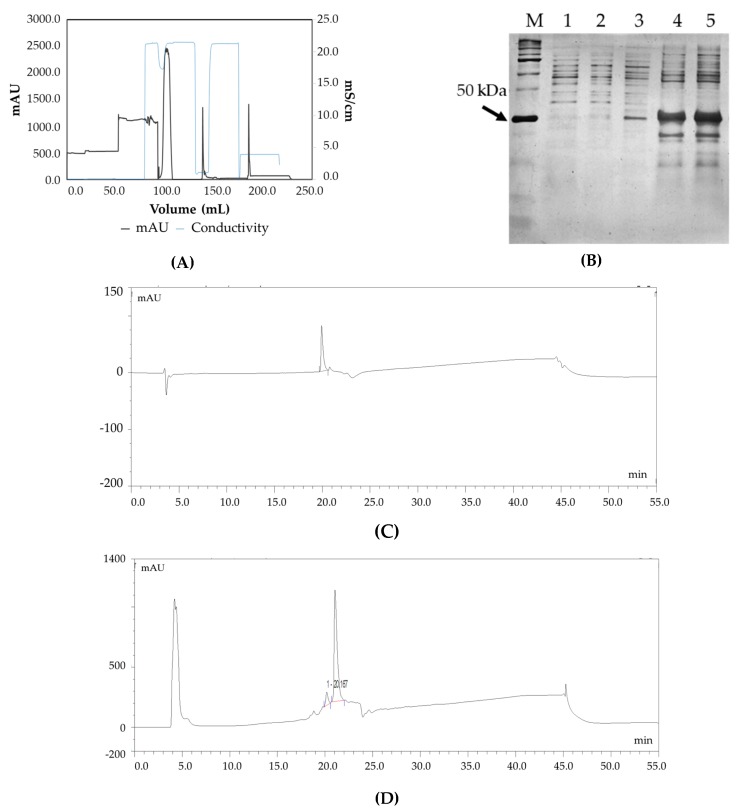
(**A**) Chromatogram of preparative scale purification of the Fab using Capto L affinity chromatography: absorbance (black trace) and conductivity (grey trace) (**B**) Confirmation of Fab molecule by SDS PAGE. Lane M: Protein Marker, Lane 1: Uninduced, Lane 2: Uninduced, Lane 3: Periplasmic extract, Lane 4: Concentrated periplasmic extract, Lane 5: Concentrated periplasmic extract (**C**) RP-HPLC chromatogram of the standard molecule, (**D**) RP-HPLC chromatogram of Fab molecule.

**Figure 3:**
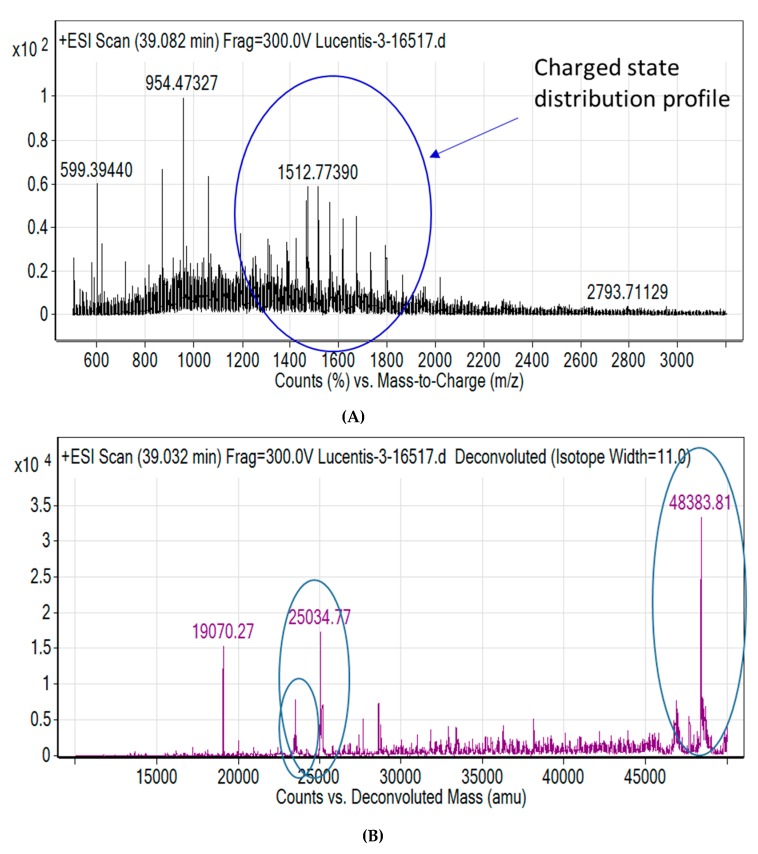
The LC/MS analysis of fab (**A**) Mass spectrum (**B**) De-convoluted mass spectrum. The circled peaks show the parent molecules, heavy chain and light chain respectively.

Capto L^TM^ affinity chromatography was used to purify the target Fab (Figure 2A). The purified protein had a yield of more than 85%. This was confirmed by SDS PAGE analysis where a prominent band was observed at 48 kDa (Figure 2B). Figure 2C,D illustrate the RP-HPLC chromatogram of the Fab standard and the Fab produced. The quantification was achieved by comparing the peak areas of the standard protein (known concentration) with that of the target protein in the periplasm extract (pre-filtered) based on identical retention times. Figure 3A shows the charge distribution profile of the intact Fab with a maximum charge of +32 based on LC/MS analysis. Upon deconvolution by the maximum entropy algorithm, the intact mass obtained was 48383 Da (Figure 3B) which is in agreement with the theoretical mass of the Fab. In addition, masses of 19 kDa and 25 kDa were also seen, corresponding to the light chain and heavy chain of the Fab, respectively. Thus, the analysis was able to confirm the product formation.

### 3.3. Fab Quantification Using BLI

**Figure 4:**
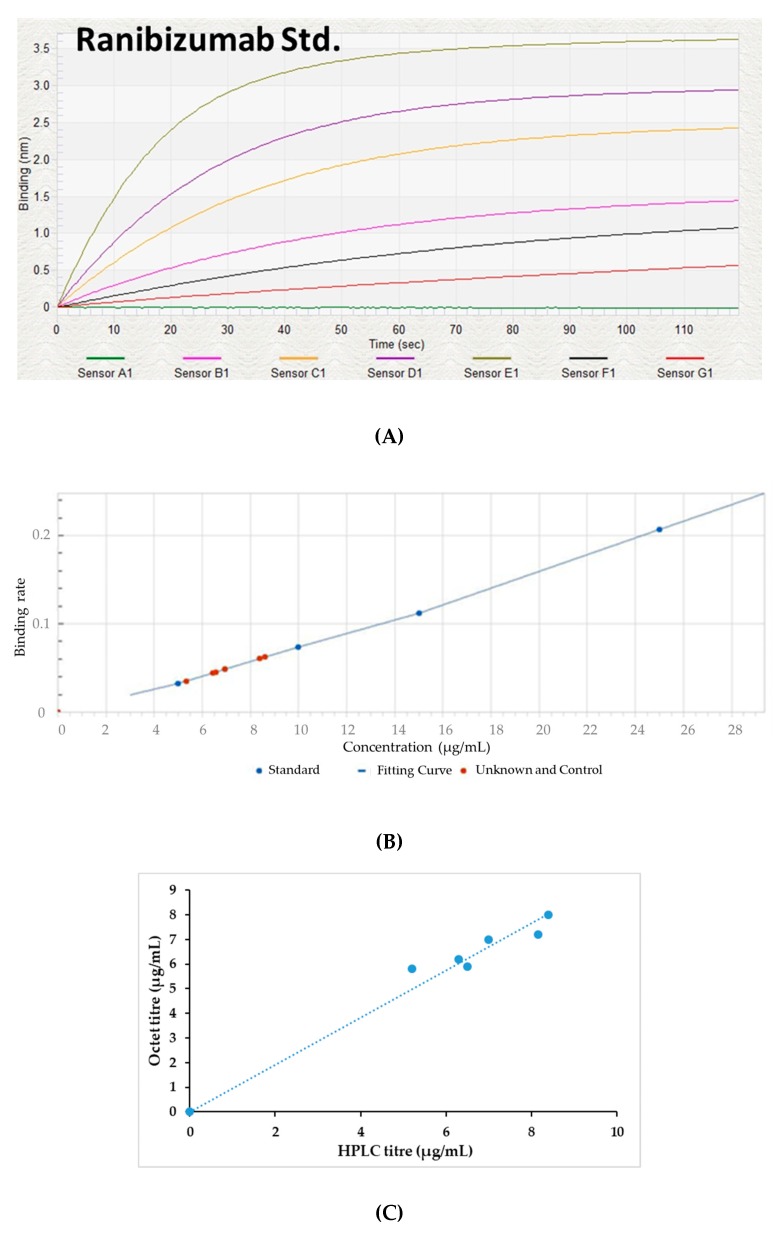
(**A**) The real-time binding data of the standard Fab molecule, (**B**) a graph depicting the binding rate of the standard as well as target Fab protein, (**C**) the comparison of Fab protein measured by Octet and RP-HPLC methods.

Figure 4A,B present the binding rate curve of the standard molecule as well as expressed target protein. The reported value of the assay sensitivity for Fab fragment quantification was in the range of 0.05–300 µg/mL. The quantification results of Fab obtained using HPLC were compared and BLI HPLC was performed using the samples without any dilution. Differences between the titer values obtained using the two methods were less than 10%, which indicates the comparability between the two (Figure 4C).

### 3.4. Effect of Process Parameters

#### 3.4.1. Effect of Temperature

**Figure 5:**
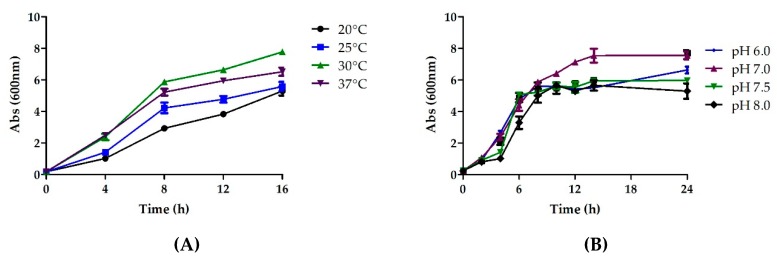

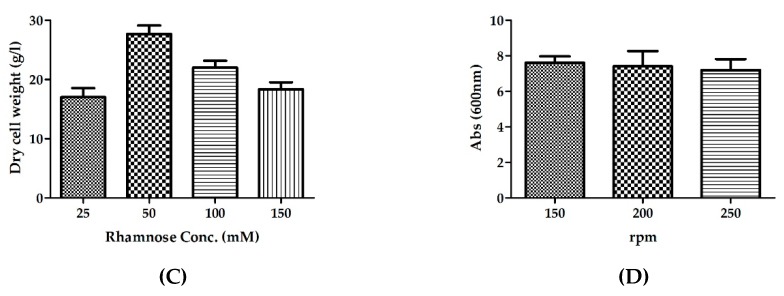
The optimization of various physiochemical parameters to enable higher biomass production, (**A**) temperature (**B**) pH (**C**) inducer concentration and (**D**) agitation.

Once a desired chemically defined medium had been chosen, the next step was to look at the various growth co-factors, both physical and physiochemical. This was done in order to determine the contribution of each parameter during Fab production. The temperature during a fermentation process is known to be a critical parameter as it affects plasmid stability, cellular metabolism and growth, and protein solubility [33,34]. The literature suggests that fermentation at temperatures below 37 °C improves the quality and folding of the recombinant proteins and results in the reduced formation of byproducts and metabolic heat [35]. The biomass production was studied at different incubation temperatures ranging from 20 to 37 °C by incubating the cultures at 200 rpm. Cultivation at 30 °C for 12 h in a shake flask led to the highest OD of 10.27 (Figure 5A). Thus, 30 °C was selected as the optimum temperature for cultivation.

#### 3.4.2. Effect of pH

Another important physiological parameter that significantly impacts cell growth and protein stability is the pH of the medium [33]. Shake flask study was performed at pH levels of 6.0, 6.5, 7.0, 7.5, and 8.0. Maximum OD of 8 was achieved at pH 7.0, which was chosen as the optimum pH for further studies (Figure 5B).

#### 3.4.3. Effect of Inducer Concentration

Selecting a suitable expression system is crucial for achieving high productivity, plasmid stability, and desirable expression [36,37,38]. The L-rhamnose-inducible promoter is capable of expressing soluble recombinant proteins that are susceptible to insoluble protein formation at high concentrations [39]. Protein expression was induced with 25–150 mM rhamnose after 8 h of secondary culture or when OD of *E. coli* culture is 8. Cultures were grown at 30 °C for an additional 8 h. The highest biomass was observed in the culture induced with 50 mM rhamnose compared to that obtained with the other induction levels (Figure 5C).

#### 3.4.4. Effect of Agitation

Agitation is required to achieve homogenous mixing, thus ensuring uniform availability of oxygen, nutrients, additives, and necessary substrates to all cells [40]. In order to determine the optimum agitation speed for our culture, protein expression was evaluated at different agitation conditions (150, 200, 250 rpm) and protein expression was induced by supplementing 50 mM rhamnose at culture OD_600 nm_ 6.0. Similar biomass production was observed at all three levels tested (Figure 5D). Thus, for subsequent experiments, 200 rpm was chosen for bacterial cultivation.

### 3.5. One Factor at a Time (OFAT) Experimentation for Screening of Media Components

To evaluate the impact of the various additives, the OFAT approach was used to screen the factors before performing a DOE. Experiments were performed at 30 °C, pH 7.0, 200 rpm, and with 50 mM rhamnose. The effect of 25 different additives (e.g., a carbon source, nitrogen source, vitamins, and buffering agents) were examined and the Fab titer was measured by HPLC. Additives yielding recombinant protein concentration greater than 8.0 mg/L were considered to have a significant effect. This was chosen with respect to the basal Riesenberg medium with no additives which gave a titer of 8.0 mg/mL. Based on these lines, of the 25 additives examined, 8 nutrients (sucrose, potassium sulfate, calcium sulfate, dipotassium phosphate, biotin, pantothenate, cyanocobalamin, glutathione) were found to have the highest impact on Fab production (Table 3). Additionally, a higher purity (more than 80%) was observed with these eight additives.

An optimum source of carbon is crucial to attaining the desired productivity in the culture process. The addition of excess glucose in the culture media for the desired biomass production would result in the reduction in pH due to the formation of the acetic acid and this which will be detrimental to the product quality and also for controlling the pH in shake flasks. This issue can be resolved using sucrose instead of glucose. Besides being a rich source of energy, sucrose aids in the periplasmic expression of the protein due to the enhanced folding of proteins and osmotic enlargement of the periplasmic space [9]. This is consistent with the results of our study, in which sucrose resulted in the enhanced productivity of the Fab molecule (83% higher compared to basal medium) (Table 3).

Maintaining the medium pH is necessary to achieve high productivity and protein stability. Potassium sulfate and dipotassium phosphate, being buffering agents, enhance the ability to maintain the desired pH of the medium. Hence, while a higher concentration of potassium sulfate increased productivity by 53% as compared to the basal medium, the addition of dipotassium phosphate resulted in a 137% higher productivity (Table 3). 

The sulphate ion in bacterial cells is readily utilized upon entering the cell by active transportation wherein it is activated by the ATP sulfurylase enzyme [41]. This explains the 52% increase in productivity upon increasing calcium sulfate (0.2%) (Table 3).

*E. coli* is known to achieve better growth in media that has been supplemented with vitamins and co-factors [42,43]. These co-factors participate in multiple processes including but not limited to enzymatic reactions, transcription, translation, and signal transduction. This is why many researchers [42,43] have demonstrated that the use of co-factors accompanied by NADH/NAD+ in cellular metabolic reactions results in higher biomass (43% higher compared to basal medium) (Table 3). Pantothenate, the other effective factor from our results, is a carrier of the acyl group which plays an important role in the formation of coenzyme A [42]. Another cofactor that was found to have a significant impact is biotin, as it is involved in the transport of CO_2_ [43]. During the ATP-dependent carboxylation of acetyl-CoA, the biotin carboxyl carrier protein is part of the acetyl-CoA carboxylase that serves as a carrier protein in the malonyl CoA formation pathway [43]. In our study, the addition of biotin enhanced biomass production by 73% compared to the basal medium without biotin (Table 3).

Glutathione, a well-known antioxidant, helps in maintaining the balance between reduction and oxidation potentials by regulating interconversion of the reduced and oxidized forms (e.g., NAD(P)H/ NAD(P)+) of redox molecules [44]. It also helps to reshuffle incorrectly formed disulfides. Its higher concentration resulted in a 190% increase in productivity compared to the basal medium (Table 3).

### 3.6. Screening of Additives using Fractional Factorial DOE 

**Figure 6:**
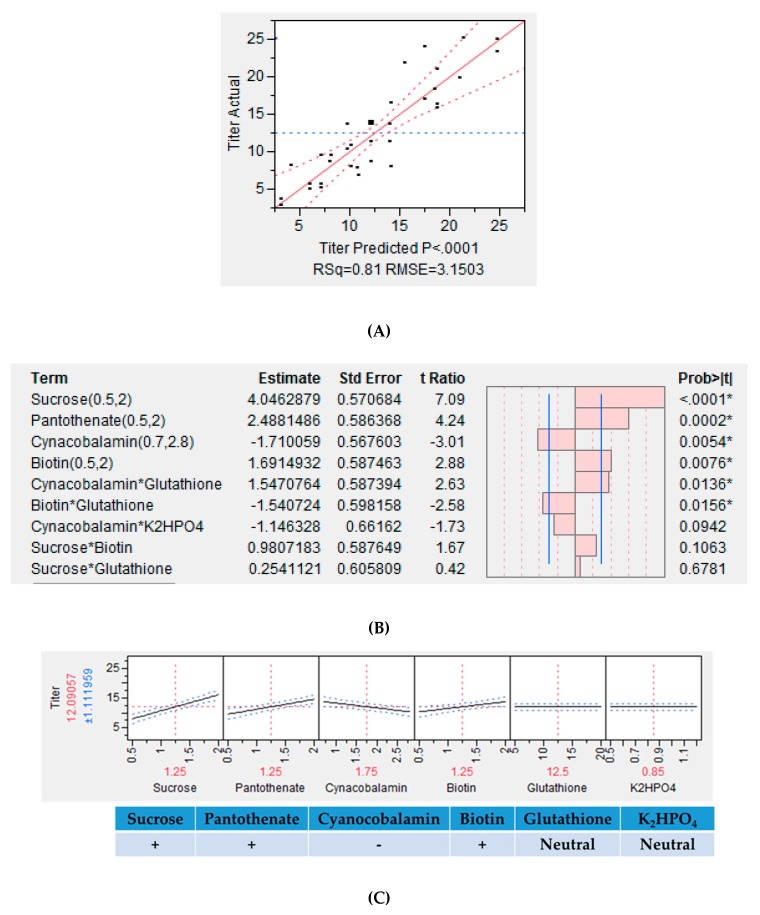

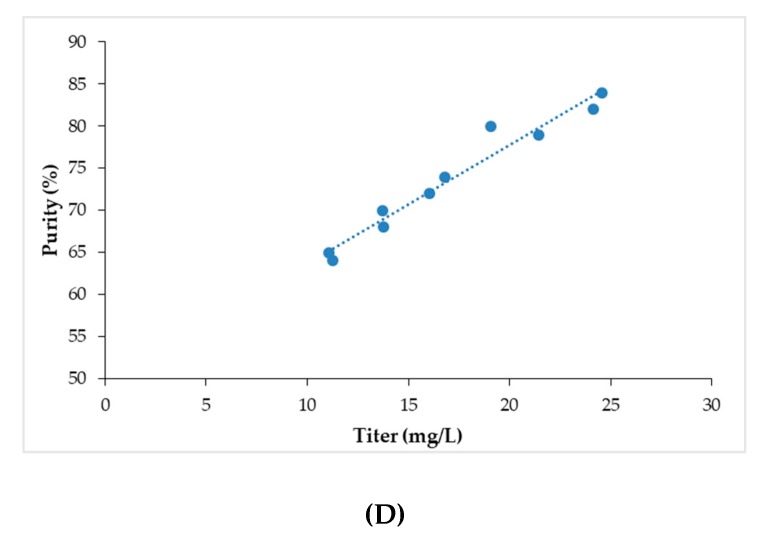
The results of the design of experiment (DOE) study showing (**A**) the actual vs. predicted plot for Fab production; (**B**) the estimates of the interactions amongst the sorted parameters, * *P* < 0.05; (**C**) the prediction profiles for Fab production; (**D**) the comparison of Fab titer vs. purity.

Statistical analysis of the data was performed as per the procedure described earlier. Media components found to have a significant impact on Fab titer and purity, as estimated by HPLC, were used to build a model. Carbon source, vitamins, buffering agents, and additives such as sucrose, potassium sulfate, calcium sulfate, dipotassium phosphate, biotin, pathothenate, cyanocobalamin, and glutathione were examined in this investigation. The resulting model is illustrated in Figure 6A. It is evident that the model is statistically relevant (R^2^ = 0.81, RMSE = 3.15). Sucrose, panthothenate, cyanocobalamin, K_2_HPO_4_, biotin, and glutathione are seen to directly impact Fab production (Figure 6B). The results are in agreement with those from the OFAT study. Among the eight factors identified in the OFAT study that were further examined through fractional factorial DOE, only six were found to have a significant effect on Fab production. While concentrations of sucrose, panthothenate, and biotin are positively correlated to mAb yield, the concentration of cyanocobalamin is negatively correlated. Sucrose had the highest positive impact on mAb yield. Additionally, a significant level of interaction was observed between glutathione and K_2_HPO_4_ and an appreciable Fab production was observed even at their lower concentrations (Figure 6C). Other significant interactions among the media components included that of glutathione with cyanocobalamin (cyanocobalamin-glutathione) and biotin (biotin-glutathione) (Figure 6B). Figure 6D depicts the correlation between purity and titer levels and it is seen in the application under consideration that we are able to achieve higher purities at higher titer values. 

## 4. Discussion

Fermentation is arguably the most vital step in the process of biotherapeutic production. Optimization of media components and process parameters are crucial for achieving optimized productivity without compromising on product quality. Owing to the dynamic nature of the various phases of bioprocesses, the quality of the product primarily depends on the microenvironment of the cell, which in turn is determined by the design of fermentation medium as well as the process conditions. In light of this, two aspects require due consideration: the role of each media component in microbial growth and the interactions among the components. However, it is not feasible to study all possible concentrations and combinations of the myriad components that are typically present in the media. In this study, we have attempted to identify and optimize the critical media components with the minimum number of experiments, as suggested in the QbD paradigm. The results provide an insight into the effects of the various inorganic compounds and other media additives towards maximizing production of an antibody fragment biotherapeutic. These findings could serve as the foundation for creating a platform process for the manufacturing of such products in *E. coli*.

Besides the identification of the optimal medium, the significant role of process parameters (temperature, pH, agitation) on cell growth and product quality has also been demonstrated in this study, complementing already published research [45]. QbD-based media development was performed to achieve greater process understanding so as to achieve optimal product titer and quality. Besides media composition, certain process parameters too are highly critical for the protein production process. One such factor that has been identified is temperature. Operating at a temperature of 30 °C resulted in optimal protein production in our case, both in terms of quality and quantity. On similar lines; pH and agitation too have contributing roles during protein production. These were studied and tuned to attain optimum yield and quality.

The inducer concentration and induction period are other factors that affect protein yield. Just as low inducer levels result in suboptimal induction and low recombinant protein yields in the higher concentration of inducer results in the formation of protein aggregates and protein toxicity, consequently diminishing the productivity levels [46]. The crucial role of inducers is well substantiated by the fact that out of all the inducer concentrations studied, the highest biomass was attained particularly with 50 mM rhamnose at 30 °C and a pH of 7.0 when induced for 8 h.

In reality, more than one factor and nutrient source are simultaneously involved in any process, thus, making Design of experiment (DoE) based studies indispensable [47]. Results reveal that sucrose, pathothenate, biotin, and cyanocobalamin have a significant impact whereas glutathione and K_2_HPO_4_ are relatively less significant. A goodness of fit of R^2^ > 0.80 implies a satisfactory agreement between the predicted and the actual values. Media additives such as sucrose, pantothenate and biotin improved the Fab expression levels whereas a higher concentration of cyanocobalamin resulted in a reduced expression. Glutathione and K_2_HPO_4_ produced no appreciable change in the Fab expression levels. Interesting to note was the reduction in Fab levels due to the interaction between biotin and glutathione, which underlined the crucial role of interactions among multiple additives in protein production. It should be highlighted that in most cases, the purity of Fab was higher for the experiments when the titer was higher. 

Implementation of the QbD approach has become inevitable in order to be able to identify and optimize crucial media components as well as determine the essential interactions amongst them. Thus, this paper presents the results from an effort to systematically study the impact of media components and process parameters for the production of a Fab fragment. The growth medium was rapidly optimized with the aid of the statistical experiment design. The highest fab yield in this study was 25 mg/L, which is a significant yield compared to published reports. The benefits of following a QbD based approach is well demonstrated.

## Figures and Tables

**Table 1:** The key attributes of different media that were screened for Fab production.

Medium	Components
Cossins Medium	(NH_4_)_2_SO_4_ (5.2 g/L), NaH_2_PO_4_ (4.15 g/L), KCl (4.025 g/L), Citric Acid (5.2 g/L), Glycerol (93.33 g/L), MgSO_4_.7H_2_O (1.04 g/L), Thiamine Hydrochloride (6.67 mM) and Trace Elements *
Sletta Medium	KH_2_PO_4_ (16.6 g/L), (NH_4_)_2_HPO_4_ (4.0 g/L), Citric Acid (2.1 g/L), Glucose (20 g/L), MgSO_4_.7H_2_O (1.04 g/L) and Trace Elements *
King Medium	(NH_4_)_2_SO_4_ (5.2 g/L) NaH_2_PO_4_ (4.15 g/L) Yeast Extract (5 g/L), Glycerol, MgSO_4_.7H_2_O (1.04 g/L) and Trace Elements *
Zhang Medium	Glycerol (20 g/L), KH_2_PO_4_ (20 g/L), (NH_4_)_2_HPO_4_ (5 g/L), Na (Pyruvate) (5 g/L), MgSO_4_.7H_2_O (1.04 g/L), Thiamine Hydrochloride (6.67 mM) and Trace Elements *
Champion Medium	(NH_4_)_2_SO_4_ (55.7 mM), NaH_2_PO_4_ (13.9 mM), K_2_HPO_4_ (21.9 mM), Sodium Citrate (5 mM), KCl (29.6 mM), MgSO_4_.7H_2_O (14.7 mM), NZ Amine AS (1.11%), Yeast Extract (1.11%), Glucose (0.11%) and Trace Elements *
Riesenberg Medium	Glucose (27.5 g/L), KH_2_PO_4_ (13.3 g/L), (NH_4_)_2_HPO_4_ (4.0 g/L), Citric Acid (1.7 g/L), MgSO_4_.7H_2_O (1.04 g/L), Thiamine Hydrochloride (4.5 mg/L) and Trace Elements *

* Trace elements include Na-EDTA, MnCl_2_, CoCl_2_, CuCl_2_, H_3_BO_4_, Na_2_MoO_4_, Zinc acetate, and Ferric ammonium citrate.

**Table 2:** The assigned concentrations of variables at different levels in the Fractional factorial design for Fab production.

S. No.	Variables	Lower Level	Higher Level
1	K_2_HPO_4_ (%)	0.5	1.2
2	Biotin (mg/L)	0.5	2
3	Glutathione (mM)	5	20
4	Sucrose (%)	0.5	2
5	Potassium Sulfate (%)	0.09	0.36
6	Calcium Sulfate (%)	0.05	0.2
7	Panthothenic Acid (mg/L)	0.5	2
8	Cyanocobalamin (mg/L)	0.7	2.8

**Table 3:** The protein titre obtained as a result of the addition of various nutrient additives as found by the one factor at a time (OFAT) approach.

S. No.	Media Additives	Concentration	Protein Titre (mg/L)
1	CoSO_4_ (g/L)	0.75	1.24
2	Di-sodium succinate (%)	1.00	2.10
3	NaEDTA (mg/L)	10.50	2.21
4	Nickel sulphate (%)	0.25	2.35
5	Al_2_(SO_4_)_3_ (g/L)	2.00	2.75
6	NaCl (%)	1.00	3.34
7	Yeast extract (%)	1.00	4.50
8	Peptone (%)	2.00	4.79
9	Glycine (%)	0.20	4.90
10	NH_4_CI (%)	0.20	5.22
11	FeSO_4_ (%)	0.10	5.73
12	(NH_4_)_2_SO_4_ (%)	0.40	5.95
13	L-Isoleucine (g/L)	0.20	6.75
14	FeCl_2_ (%)	2.70	6.95
15	L-Proline (g/L)	0.20	7.30
16	NaHCO_3_ (%)	1.00	7.65
17	ZnCl_2_ (%)	0.20	7.80
18	Panthothenic Acid (mg/L)	1.00	8.30
19	Calcium sulfate (%)	0.10	8.86
20	Potassium sulfate (%)	0.18	8.88
21	Biotin (mg/L)	1.00	10.05
22	Sucrose (%)	1.00	10.65
23	Cyanocobalamin (mg/L)	1.40	12.54
24	K_2_HPO_4_ (%)	0.12	13.80
25	Glutathione (mM)	10.00	16.80
26	Control (no additive)	-	8.00
